# Measured, opportunistic, unexpected and naïve quitting: a qualitative grounded theory study of the process of quitting from the ex-smokers’ perspective

**DOI:** 10.1186/s12889-017-4326-4

**Published:** 2017-05-11

**Authors:** Andrea L Smith, Stacy M Carter, Sally M Dunlop, Becky Freeman, Simon Chapman

**Affiliations:** 1 Centre for Values, Ethics and the Law in Medicine, School of Public Health, University of Sydney, Sydney, NSW 2006 Australia; 2 Cancer Screening and Prevention, Cancer Institute NSW, Eveleigh, NSW 2015 Australia; 3 Prevention Research Collaboration, School of Public Health, The University of Sydney, Sydney, NSW 2006 Australia; 4 School of Public Health, The University of Sydney, Sydney, NSW 2006 Australia

**Keywords:** Qualitative, Grounded theory, Smoking cessation, Catastrophe theory, Stages of change, Dual process theory

## Abstract

**Background:**

To better understand the process of quitting from the ex-smokers’ perspective, and to explore the role spontaneity and planning play in quitting.

**Methods:**

Qualitative grounded theory study using in-depth interviews with 37 Australian adult ex-smokers (24–68 years; 15 males, 22 females) who quit smoking in the past 6–24 months (26 quit unassisted; 11 used assistance).

**Results:**

Based on participants’ accounts of quitting, we propose a typology of quitting experiences: measured, opportunistic, unexpected and naïve. Two key features integral to participants’ accounts of their quitting experiences were used as the basis of the typology: (1) the apparent onset of quitting (gradual through to sudden); and (2) the degree to which the smoker appeared to have prepared for quitting (no evidence through to clear evidence of preparation). The resulting 2 × 2 matrix of quitting experiences took into consideration three additional characteristics: (1) the presence or absence of a clearly identifiable trigger; (2) the amount of effort (cognitive and practical) involved in quitting; and (3) the type of cognitive process that characterised the quitting experience (reflective; impulsive; reflective and impulsive).

**Conclusions:**

Quitting typically included elements of spontaneity (impulsive behaviour) and preparation (reflective behaviour), and, importantly, the investment of time and cognitive effort by participants prior to quitting. Remarkably few participants quit completely out-of-the-blue with little or no preparation. Findings are discussed in relation to stages-of-change theory, catastrophe theory, and dual process theories, focusing on how dual process theories may provide a way of conceptualising how quitting can include elements of both spontaneity and preparation.

**Electronic supplementary material:**

The online version of this article (doi:10.1186/s12889-017-4326-4) contains supplementary material, which is available to authorized users.

## Background

Like many difficult-to-change health behaviours, the process of quitting smoking is complex and often unsuccessful. Although wanting to quit is a necessary condition for attempting to quit, it is not in itself sufficient to ensure success. We know the vast majority of smokers express regret at ever having started to smoke [1], that most smokers want to quit, and that every year about half attempt to quit [2], yet annually only 3–5% of smokers successfully quit for at least 12 months [3, 4].

Clinical practice guidelines and telephone quit-lines in countries such as Australia generally advise smokers that their chances of quitting successfully will improve if they plan their quit attempt in advance. Smokers are advised to set a quit date, address perceived barriers to quitting, seek social support, use pharmacological or behavioural support, and practice strategies to deal with cravings to smoke [5–7]. However, in 2005 the widely held belief that planning is a necessary prerequisite for quitting was challenged when a Canadian GP reported more than half of the smokers and ex-smokers she had interviewed had quit or attempted to quit without any pre-planning [8]. This finding was subsequently supported by studies in the UK [9–11], USA [12], and Sweden [13]. Several of these studies also reported that spontaneous quit attempts were more successful than planned quit attempts [8–10, 12, 13]. In contrast, several International Tobacco Control (ITC) studies reported that neither prior consideration nor delay between decision to quit and implementation was clearly related to quitting success and that there was no clear benefit of planning on short-term (1 month) cessation outcomes [14, 15]. Interestingly, a recent qualitative study has highlighted the difficulties involved in measuring concepts such as planning and spontaneity in relation to quitting, and the limitations of questionnaire-based surveys when assessing the prevalence and impact of planning on quitting success [16].

### Aim and scope of the grounded theory study

Qualitative research has the potential to make a significant contribution to our understanding of the process of quitting by offering deep insights into the experiences of smokers when they quit. Grounded theory is a qualitative methodology that has already been used to better understand processes involved in difficult-to-change health behaviors [17]. For example, grounded theory studies in the UK have provided valuable insights into why clients seek professional treatment for drinking problems, which lead to the development of a model of the behavior-change process while utilizing these services [18, 19].

In this paper we report on a grounded theory study using in-depth, one-on-one interviews with recent ex-smokers (quit >6 months but <24 months). The current study is part of a larger qualitative study exploring how and why many smokers in Australia quit without using assistance despite pharmaceutical and professional smoking cessation assistance being affordable and widely available. It is anticipated the results of this study could provide rich information about the complex and highly variable process of quitting. It is hoped this information could inform a more nuanced response to the challenge of smoking cessation perhaps, for example, by providing campaign developers with insights that might allow them to develop more targeted quit campaigns tailored to the needs of specific audiences. Our purposive sampling strategy initially focused on ex-smokers who had quit without pharmacological or professional assistance as this was our primary area of interest and is an understudied area of research [20]. We subsequently expanded our sampling to include smokers who had used assistance to quit to allow us to make analytical comparisons across cases and conditions. Our initial analysis indicated that there were more similarities than differences between the two methods of quitting and that using assistance appeared to be only one of many parts of a complicated process. In the initial analysis we also noticed very few participants appeared to have quit spontaneously (i.e. without any planning or preparation). This was noteworthy as this contradicts what many quantitative, survey-based studies into spontaneity and quitting have reported. Based on our initial findings, our subsequent analysis examined: (1) the process of successful quitting from the recent ex-smokers’ perspective; and (2) the concepts of spontaneity and planning in the participants’ accounts of quitting.

## Methods

### Rationale for choice of methodology

A constructivist grounded theory methodology underpinned the study design, research questions, data collection, analysis and interpretation [17]. Grounded theory was established in 1967 to reinstate inductive field-work underpinned by interactionist sociological theory. [21] Grounded theory has evolved considerably since then and is now one of the most-used methodologies in qualitative research, including health. In this current study we have drawn on the work of Kathy Charmaz, a contemporary leader in the field of grounded theory. Charmaz’s constructivist grounded theory methodology is ideally suited to studying processes in individuals such as the process of quitting smoking (see Table 1 for key characteristics of a grounded theory study) [17].

**Table 1:** Key characteristics of a grounded theory study [17, 44]

• In a grounded theory study, theory is generated rather than tested.
• Data collection and analysis are cyclical and take place throughout the study.
• The sampling strategy (and sample size) is not pre-determined but is instead flexible.
• Recruitment continues until the central concepts in the developing theory are well understood (i.e. theoretical saturation is reached).
• Analysis typically involves:
(1) coding, in which the researcher develops codes to specify elements of the process under study
(2) memoing, in which the researcher writes analytical memos exploring how elements in the process under study relate to one another and the range of variation in the process
(3) diagramming or modeling, in which the researcher maps the relationships between elements in the process under study. As analysis progresses, data collection and analysis become more focused on clarifying and relating an ever-decreasing number of central concepts.

Our methods were also influenced by informed grounded theory rather than Glaser’s classic grounded theory [21–23]. Informed grounded theory recognizes that pre-existing theories can help the researcher to focus attention on certain phenomena, aspects or nuances. Pre-existing theories can provide a framework for thinking about a problem and for seeing beyond the data [24]. In this current study we were mindful of theories relating to how people think, how they make decisions, and how their motivational system generates action [25]. We were also aware of behaviorist theories [26, 27], rationality-based cognitive theories [28], catastrophe theory [9], comprehensive theories of addiction such as PRIME (plans, responses, impulses, motives and evaluations) theory of motivation [29], and theories of hard-to-maintain behavior change such as CEOS (context, executive and operational system) dual process theory [30].

### Recruitment and participant selection

We recruited participants from the general community using traditional media (media release, print and online newspaper articles, talk-back radio) as well as social media. Eligible participants were former smokers who had quit in the previous 6 months to 2 years. Risk of relapse to smoking, which reduces with time quit [31, 32] was balanced against potential for recall bias [33]. Participants were classified as having quit unassisted or with the help of pharmacotherapy or professionally mediated behavioural support (see [34] for full definition of unassisted and assisted).

Each participant was asked about their smoking and quitting histories (e.g. cigarettes per day, years of smoking, number and type of prior quit attempts, use of assistance to quit) and to provide basic demographic information (e.g. age, gender, education, income and geographical location). In keeping with grounded theory methodology, sampling evolved from a purposive to a theoretical strategy as the study progressed [17]. Purposive sampling allowed us to interview participants with varied smoking and quitting histories from a diverse range of backgrounds. This sampling strategy ensured we generated rich, relevant and diverse data pertinent to the research questions. As data analysis progressed we moved to theoretical sampling in order to test our evolving theories about the process of successful quitting. Participants were offered AU$80 reimbursement for their time.

### Data collection

We interviewed 37 Australian adult (18+ years) former smokers who had quit within the past 6 months to 2 years ( Table 2 ). Interviews took place between December 2012 and December 2015. Participants nominated to be interviewed face-to-face or by telephone. All interviews were conducted by AS. The University of Sydney Human Research Ethics Committee approved all study procedures and materials (reference number 15019). Participants provided written consent for their participation prior to enrolment in the study. Pseudonyms were used to ensure anonymity.

**Table 2:** Demographic, smoking and quitting characteristics of participants

Characteristic	Participants (*n* = 37)
Gender	
Male	15
Female	22
Age (years)	
20–29	4
30–39	6
40–49	9
50–59	11
60–69	7
Geographical location^a^	
Major cities	25
Inner regional Australia	4
Outer regional Australia	7
Remote Australia	1
Total household income (AU$)^b^	
≤ 30 K	7
> 30 K–60 K	5
> 60 K–90 K	6
> 90 K–120 K	7
> 120 K	9
Cigarettes per day	
< 10 CPD	11
> 10 CPD	26
Use of assistance to quit	
Used assistance	11
Unassisted	26
Previous quit attempts	
None	3
< 3	16
3–10	11
> 10	7
Previous experience of assistance	
Had never tried to quit before	3
Had never used assistance to quit	11
Had previously used assistance to quit	23

^a^Classified according to the Australian Standard Geographical Classification Remoteness Area system

^b^3 participants did not answer the question on income

A semi-structured interview guide was used for each interview. Participants were asked to talk about their smoking and quitting from when they first started to smoke. A timeline was drawn up of their smoking and quitting history on which all quit attempts were documented. Questions evolved as recruitment and interviewing progressed, with questions in later interviews becoming more specific in order to further develop provisional ideas and theories. The screening questionnaire (Additional file 1) and interview guide (Additional file 2) were pilot tested prior to study commencement. Interviews were audio-recorded and transcribed verbatim. Interviews lasted between 37 min and 2 h 15 min. Field notes were made directly after each interview. Data analysis from each interview helped to inform subsequent sampling, allowing us to target who to interview next and what questions to ask them. This purposive and then theoretical sampling allowed us to test the validity and relevance of the proposed typology of quitting experiences. It also allowed us to be confident that our sampling had been adequate, that is we had continued to collect data until we could fully explain how the key elements in the quitting process related to one another and that our theory explained the variation in the experiences of quitting as reported by participants.

### Coding and analysis

We used the computer-assisted qualitative data analysis software NVivo 10 (QSR International) for data management and coding. Coding and memoing were carried out by AS, a trained and experienced qualitative researcher. Interview transcripts were read several times before being coded line-by-line (open coding) [17]. The line-by-line coding aimed to identify what was important to that particular participant when they quit. Comparison of the line-by-line codes from within individual interviews and across all interviews lead to a consolidation and refinement of codes based on patterns observed across interviews relating to key circumstances surrounding quitting (focused coding). Coding was followed by diagramming and modeling to establish how various elements in the quitting process were related to one another.

To improve validity of this interpretive study, the open and focused codes, the coding hierarchy, the memos, the diagrams and models, and the developing ideas and theories were regularly discussed among members of the research team, whose expertise in smoking cessation, behavioural psychology, public health ethics and qualitative health research methodology were critical to the interpretation of the data. These discussions fostered a deeper understanding of the data and ensured our conclusions were grounded in the data. Transparency and auditability of the analytical process were enhanced through the use of memos that documented the researchers’ provisional interpretations.

## Results

### Overview: the process of quitting

Participants were initially divided into those who took a slower, less direct path to quitting success (slow quitters) and those who quit rapidly, suddenly, and in some cases almost unexpectedly (fast quitters). However, we suspected the fast and slow dichotomy was too simplistic and did not fully capture the variation and complexity of participants’ quitting experiences. This suspicion was confirmed when we attempted to divide participants into those whose experience of quitting had been slow and less direct and those whose experience had been one of quitting rapidly or suddenly. We found that not all participants’ quitting experiences could be clearly classified as being slow or fast; indeed it appeared as if many participants’ experiences included elements of fast and slow quitting. At this point we went back to the coded interview transcripts, field notes and memos to see if further analysis could create a typology that more closely reflected the process of quitting as described by participants.

**Fig. 1:**
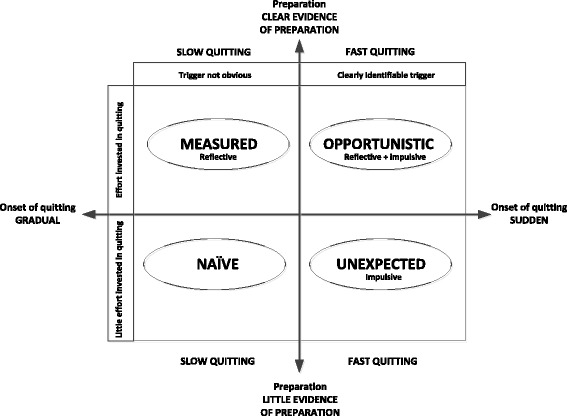
A typology of quitting experiences*.* The experience of quitting broadly appears to be fast or slow, but can be further classified according to a number of criteria: the apparent onset of the quit attempt (gradual through to sudden); evidence of preparation (clear evidence through to little or no evidence); the amount and type of cognitive effort involved in the quit attempt (reflective only, impulsive only, or both reflective and impulsive); and whether quitting was triggered by a specific event (clearly identifiable trigger through to no clearly identifiable trigger)

After memoing and modeling various possibilities we concluded that the range and complexity of participants’ experiences could be accounted for if each participant’s quit attempt and their quitting history were assessed against two criteria: (1) the apparent onset of quitting (gradual through to sudden); and (2) the degree to which the smoker appeared to have prepared for quitting (no evidence of preparation through to clear evidence of preparation). Combining the onset of quitting and preparation for quitting produced a 2 × 2 matrix (Fig. 1) in which fast and slow quitting were sub-categorised, resulting in a typology of four quitting experiences: measured, opportunistic, unexpected and naïve. Importantly, this matrix also took into account three other factors that varied among participants: (1) the presence or absence of a clearly identifiable trigger; (2) the amount of cognitive effort involved in thinking about quitting; and (3) the type of cognitive process that drove the quit attempt (reflective; impulsive; or reflective and impulsive).

**Fig. 2:**
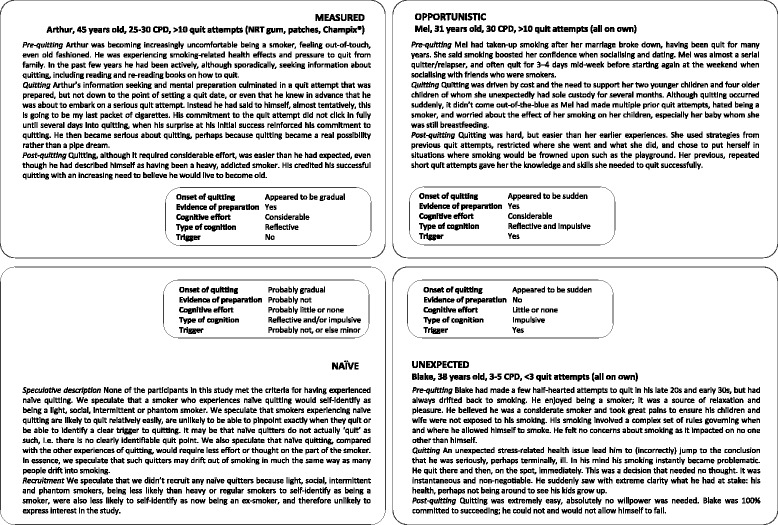
Illustrative case studies of the four quitting experiences: measured, opportunistic, unexpected and naïve

Each participant’s account of quitting was then reviewed by returning to their interview transcript, field notes and memos. In so doing we satisfied ourselves that the typology accounted for the range of quitting experiences reported by all 37 participants (see Fig. 2 for illustrative case studies of each quitting experience). Within the study sample we observed accounts of measured, opportunistic and unexpected quitting. However, despite continuing to sample theoretically for participants who may have been naïve quitters, no accounts of naïve quitting were found. We comment in the discussion why this might be.

### Fast quitting: unexpected and opportunistic quitters

Unexpected and opportunistic quitters quit suddenly. Quitting was characterized by the presence of a clearly identifiable event that triggered quitting.

#### Opportunistic quitters

For opportunistic quitters, although a trigger was present, quitting had not come out-of-the-blue. Close examination of the participant’s quitting history revealed quitting had been preceded by a period of deliberation and planning.

*‘I realised that on that trip I was going to not be able to smoke on the aircraft, I was going to not be able to smoke in the hire car. Not be able to smoke in the hotels where I stayed. Wouldn't be able to smoke in the homes of my children. I thought what a perfect time to quit.’* Gregory, 68 years old, opportunistic quitter.

Opportunistic quitters leveraged their quit attempt around a particular event or set of circumstances. They had been thinking about quitting, and were ready and able to recognize and embrace an upcoming event or set of circumstances as an opportunity on which to hang their quit attempt.

*‘I said to my boyfriend and I said to myself, it's like the day I go [relocate for 3 months] that's the day I'm going to quit because it's going to be easier, that's probably the easiest option I've got there to quit. I thought right that's a chance there to quit, so I took it.*’ Sarah, 26 years old, opportunistic quitter.

Not all opportunistic quitters had set a quit date or chosen a significant event around which they planned to quit. Instead, some opportunistic quitters quit almost on impulse in response to a situation or a coming together of events that suddenly represented an opportunity too good to miss. It was as if they had been waiting for the right moment, and having already invested time and effort into thinking about quitting, and into making quitting personally important, they were ready and able to recognize and seize an opportunity to quit when it presented itself.

*‘I certainly was thinking ahead [about quitting] … I think getting this book about it was a step and making myself accessible to the information about the negatives was also a step.’* Lesley, 58 years old, opportunistic quitter.

Although quitting arose against a backdrop of wanting or needing to quit, of having thought about quitting, and of having had an intention to quit at some point in the future, when the participant finally quit they were acting on impulse, in response to a momentary increase in their motivation brought about by social and environmental circumstances that suddenly made quitting attractive, easier or more important.

*‘But this is what serendipity threw my way. Once I had the circumstances, which were serendipitous, I certainly did make sure I used them… I was saying to myself that day … it's a really good opportunity and it's really important.’* Lesley, 58 years old, opportunistic quitter.

#### Unexpected quitters

In contrast, although unexpected quitters quit suddenly, in response to a trigger, their quitting was unplanned in that they had no prior intention of quitting and were instead acting purely on impulse. On the whole they had been ‘happy’ being a smoker or else resigned to being a smoker for life.

*‘I didn't feel like I needed to give up. I didn't want to give up …I enjoyed it. Yeah, I enjoyed the smoking. It was a relaxer.’* Blake, 38 years old, unexpected quitter.

An unexpected event, often health-related, forced the participant to take immediate stock of their smoking. This event could be described as an existential or identity threat that forced them to re-evaluate their smoking.

*‘Something was going wrong with my body and I had – I was – I thought, I had cancer. Because my father had cancer, he passed away with cancer … I thought, just literally, the moment, cancer – it was then, it was the big health thing. I wasn't immortal anymore. Whoa.’* Blake, 38 years old, unexpected quitter.

For unexpected quitters the decision to quit happened instantaneously. The participant often stated that they had no choice, that the decision to quit did not require any thought, that it had been taken out of their hands.

*‘I walked out the hospital and threw the pack in the bin. I haven’t touched them since. It just went out of my mind. I didn’t think about it.’* Patrick, 60 years old, unexpected quitter.

Unexpected quitters frequently claimed quitting was easy, requiring surprisingly little effort or willpower.

*‘It hasn't been hard. Over the last six months there's hardly been an event that's occurred where I would have wanted a fag.’* John, 62 years old, unexpected quitter.

### Slow quitting: measured and naïve quitters

Slow quitters gradually moved towards quitting success, often through a circuitous or winding route. In contrast to fast quitters, there appeared to be no specific, memorable or clearly defined trigger associated with their final successful quitting.

*‘There was no particular [trigger]… I had something in the back of my mind that I should quit. I should quit. I should quit. Then I thought one day, okay this is the last cigarette.’* Matthew, 53 years old, measured quitter.

#### Measured quitters

Quitting appeared to be driven by an acceptance that smoking was wrong and that they should quit, but this desire or need to quit appeared to be difficult to maintain and many struggled to make quitting important enough to sustain their quitting in the long-term. Measured quitters often wrestled with their desire or need to quit versus their desire or need to smoke.

*‘There was constant talk about we really should give up, but it was always a should rather than a I really want to … part of the problem was that I still enjoyed smoking … I wasn't one of those smokers who had gotten to the point of going oh this is really bad, that's really horrible. I knew it was bad for me but I still found it very pleasurable.’* Juliette, 46 years old, measured quitter.

Measured quitters often seemed to have been searching for a good enough reason to quit and to stay quit.

*‘The price had gone up, you know it was becoming more expensive and like I said [I had] less money. My health, being older, it was noticeable the increase in my smoking, and a noticeable difference in my health. There was just no reason, I couldn't talk myself into it, there was no reason to keep smoking.’* Josephine, 56 years old, measured quitter.

Many measured quitters had tried to quit before (similar to opportunistic quitters, but unlike unexpected quitters), often using a range of different strategies and techniques to prepare for and sustain their quit attempt. Many seemed to find quitting a struggle, something that required considerable effort and dedication, and an acceptance that it was not going to be easy to quit and that it might require several attempts before they succeeded. Participants who used assistance to quit were usually measured quitters (although a few were opportunistic quitters).

*‘We approached this last time not being very up about it, going well we know it's really hard and shitty at the beginning. I guess you just get more realistic. It's like doing exercise. You just get used to it, that you can fall off and getting back to it's going to be bloody hard, it will hurt, but eventually it will feel good.’* Juliette, 46 years old, measured quitter.

#### Naïve quitters

None of the participants was a naïve quitter. This category therefore remained speculative (Fig. 2). We comment in the Discussion why this might be.

## Discussion

We have created a typology that accounts for the experience of quitting as reported by all 37 participants. The typology is based on a number of characteristics seen across the different accounts of quitting. These characteristics interact to create a typology of quitting experiences: measured, opportunistic, unexpected or naïve. Three of these typologies were directly observed in participants’ accounts of quitting; the fourth (naïve) remains speculative. We hypothesise that naïve quitters are likely to have been light, social, intermittent, phantom or defensive smokers who may not have self-identified as smokers and therefore may not self-identify, once quit, as being an ex-smoker [35, 36]. It is possible such ex-smokers did not come forward in response to our recruitment strategies as they may not have considered the study relevant to them or their experience of quitting.

This typology of quitting experiences may help smoking cessation researchers better understand what spontaneity and planning mean in relation to successful quitting, concepts that have been acknowledged by some to be more complex than the way in which they are currently conceptualized [16, 37, 38]. The typology provides a new conceptual framework for understanding the process of successful quitting that accounts for: (1) how quit attempts and quitting success can be driven by rational plans *and* impulsive behavior, and (2) how the concept of planning should not necessarily be limited to the period immediately prior to the quit attempt but could be expanded to include planning learnt, left-over or carried forward from an earlier quit attempt.

In this study we found very few participants quit completely out-of-the-blue with little or no preparation or planning. For most participants quitting involved some form of pre-planning or preparation, making them measured or opportunistic quitters rather than unexpected quitters. In contrast, many other studies on quitting report that a significant proportion of smokers and ex-smokers quit without planning (37–52%) [8–13]. Several of these studies also report that spontaneous quit attempts are more successful than planned quit attempts [8–10, 12, 13]. Our findings are in line with those of a recent prospective US study of quit attempts in real-world settings which reported that although unplanned attempts were more prevalent (defining “planned” quit attempts as “attempts preceded by an intention not to smoke the next day”), planned attempts were more likely to succeed [38].

We suggest that some of the reported differences in prevalence and effectiveness of spontaneous versus planned quitting might be explained by two factors. The first is the lack of clarity surrounding what spontaneity and planning mean and the consequent difficulties inherent in measuring these concepts, an issue others have raised when attempting to understand the different results from studies into spontaneous quitting [11, 14]. We note that several studies [10, 12, 13, 39] reporting on the prevalence of planned versus unplanned quitting relied on a single question from the 2005 British Marketing Research Bureau household omnibus survey [9]. The question asked: ‘Which of these statements best describes how your most recent quit attempt started?’ to which the first response was ‘I didn’t plan the quit attempt in advance; I just did it’. It is possible the emotive Nike^®^ slogan-like phrase (‘I just did it’) may have influenced how participants responded. Smokers, like others seeking to change health-related behaviors, often see themselves or wish to be perceived as central to their success even when they have used some form of assistance [19, 40]. Furthermore, its position as the first response of eight may have resulted in a response-order effect [41]. These factors may in part explain Murray’s 2010 finding that on in-depth questioning, many of their participants who had originally responded ‘I didn’t plan the quit attempt in advance; I just did it’ had been misclassified as spontaneous quitters. Murray’s in-depth interviews revealed that these participants had either delayed their quitting or had used some form of assistance when they quit and therefore had not actually quit spontaneously [16].

The second explanation for the difference between studies is that previously researchers have tended to assume that spontaneity and planning are mutually exclusive: our findings challenge this assumption [8, 9, 12]. At first glance, a substantial proportion of our participants did indeed appear to have quit spontaneously, often in response to what was essentially a minor trigger. However on examining their smoking and quitting history it became clear that for many of these participants quitting had not come out-of-the-blue. This is in keeping with what Cooper and colleagues report, that most quit attempts were not made on the spur of the moment but were preceded by a period of serious consideration [14]. Many of the participants in the current study had invested time and effort into thinking about quitting, and some had made plans to quit. In these participants it was the exact timing or initiation of the quit attempt that was spontaneous or unplanned, not the quitting *per se*. Thus, these opportunistic quitters demonstrate that quitting can include elements of both spontaneity and planning. The presence of spontaneity and planning in the process of quitting reflects current theorizing about how people think, how they make decisions, and how their motivational system generates action. The presence of spontaneity and planning is reminiscent of Haidt’s elephant and rider metaphor [42], and Kahneman’s explanation in *Thinking, Fast and Slow* of why human beings depart in systematic ways from standard economic approaches to rationality [25].

Our analysis suggests the process of quitting involves both sudden (impulsive) and gradual (reflective) components. The existence of impulsive and reflective components lends further support to claims that behaviourist theories [26, 27] and rationality-based cognitive theories (e.g. the transtheorectical model of behaviour change, also known as stages of change or SOC) [28] only go so far in explaining hard-to-maintain behavior change such as quitting smoking [30]. For example, the SOC model assumes individuals make rational, coherent and stable plans that gradually move them closer to achieving a permanent change in their behaviour. This would mean smokers make a clear decision to quit, set a date to quit, and then act on this intention (i.e. decide, plan, implement). In the current study, the SOC model would be able to account for the behavior of measured quitters, but would not be able to account for opportunistic or unexpected quitters.

A number of researchers have already challenged the relevance of rationality-based cognitive theories such as SOC to smoking cessation [8, 9, 13, 43]. Our analysis supports parts of West’s 2005 critique of the SOC model, notably the suggestion that transition through pre-action stages is not always the norm or even necessary for successful change, that the change process is much more dynamic, heterogeneous and stimulus-driven than is implied by the model, and that the SOC model places too much emphasis on conscious decision-making [43]. In addition, the SOC model fails to take into account the strong situational determinants of behavior, and the fact that behavior change can arise from a response to a trigger even in apparently unmotivated individuals.

A number of alternatives have been proposed that take into account the unpredictable and dynamic nature of quitting and in particular the role of spontaneity in quitting. The catastrophe theory, based on chaos theory, proposes that tensions develop in systems in such a way that even small triggers can lead to sudden catastrophic changes [9]. According to catastrophe theory, quitting can take place unexpectedly without the smoker going through the slow process of cognitive shifts, quitting plans and intentions, and finally action. Instead, the catastrophe theory proposes smokers experience tension, or dissonance, about their smoking over a period of time but don’t act until a precipitating event triggers action. Although compelling, the catastrophe theory’s premise that many if not the majority of quit attempts are sudden and spontaneous and largely devoid of anticipatory planning does not fit with our typology of quitting experiences: as mentioned earlier, many participants who at first appeared to have quit spontaneously had actually invested time and effort into thinking about quitting.

Our typology of quitting experiences is perhaps more consistent with comprehensive theories of addiction such as West’s PRIME theory of motivation [29] and theories of hard-to-maintain behavior change such as Borland’s CEOS dual process theory [30]. These theories integrate both spontaneity and planning into the process of smoking cessation. Our typology of quitting experiences demonstrates explicitly what Borland has proposed that PRIME theory implicitly assumes: ‘spontaneity relates to peaks in fluctuating levels of longer term concern; that is, that “spontaneous” quit attempts are typically preceded by periods of deliberation that are not strong enough to trigger action rather than occurring completely out of the blue’ [37].

Successful quitting, like other behaviour changes, appears to be a struggle between our rational, reflective selves and our impulsive natures [25]. Most smokers know smoking is harmful, and most smokers want to quit. Yet their behavior is often at odds with what they know they should do. The current study indicates that for many of the participants quitting was characterized by a slow movement towards achieving that goal, with only a few of the participants taking an accelerated pathway triggered suddenly and unexpectedly by significant external events such as a diagnosis of a smoking-related illness. Many of the participants were instead influenced by a multitude of environmental and social factors and gradually come round to accepting that what they were doing (smoking) was at odds with what they valued or believed in (being in control, staying healthy, being a good role model). For some, this was a slow slog with multiple attempts to quit before success was achieved, others managed to opportunistically leverage their success off a timely trigger, while relatively few quit suddenly and unexpectedly when faced with an existential or identity threat.

### Strengths and limitations

We spoke directly and in-depth to successful recent ex-smokers. By allowing participants to talk freely and at length about their quitting experiences the data collection focused on what smokers perceived to be important. Data collection and analysis were not restricted to variables predetermined by the researchers or to a pre-existing theoretical framework. By recruiting ex-smokers who had quit in the previous 6 months to 2 years we balanced risk of relapse to smoking [31, 32] against potential for recall bias [33]. Approximately two-thirds of participants had quit on their own, reflecting recently reported Australian rates of smoking cessation assistance use [34].

We did not observe any naïve quitters among participants. We believe naïve quitters are likely to have been former light, social, intermittent, phantom or defensive smokers [35, 36], and may potentially have self-identified as non-smokers rather than serious or regular smokers. On quitting such smokers may not self-identify as being a former smoker, making our study irrelevant to them. In contrast, our study is likely to have appealed to former smokers who had smoked heavily or regularly and for whom quitting had been a far more significant event in their lives. Future research could explore the hypothesised category of naïve quitters to establish whether this quitting experience and its proposed characteristics exist.

## Conclusions

Quitting typically included elements of both spontaneity (impulsive behaviour) and preparation (reflective behaviour). Quitting came completely out-of-the-blue for only a few participants. Research that dichotomises spontaneity and planning may oversimplify the process of quitting; such oversimplification may account for the conflicting prevalence and effectiveness data for spontaneous versus unplanned quitting. The current analysis suggests quitting should be viewed as a gradual process influenced not only by events that happen immediately prior to quitting but also more distant events in the former smokers’ quitting history. Future research could focus on the role of planning and preparation carried forward from earlier quit attempts on the success of subsequent quit attempts, and on the importance of encouraging smokers to act on impulses to quit rather than focusing on getting smokers to make a rational decision to quit based on an evaluation of the costs and benefits of smoking and quitting.

## Additional files

Additional file 1: Screening questions. Questions used to screen potential study participants to assess eligbiligy, collect basic demographic data, and smoking and quitting data. (DOCX 84 kb)

Additional file 2: Interview questions. The interview schedule listing the type of questions asked in the semi-structured interviews. (DOC 40 kb)

## Abbreviations

CEOS: Context, executive and operational system
ITC: International Tobacco Control
PRIME: Plans, responses, impulses, motives and evaluations
SOC: Stages of change.
