# Supplementary material for: Measured, opportunistic, unexpected and naïve quitting: a qualitative grounded theory study of the process of quitting from the ex-smokers’ perspective
Source: BMC Public Health. 2017 May 11;17:430. doi: 10.1186/s12889-017-4326-4 (PMC5426051; doi:10.1186/s12889-017-4326-4)
Supplement: Supplementary file 1 — Screening questions. Questions used to screen potential study participants to assess eligbiligy, collect basic demographic data, and smoking and quitting data. (DOCX 84 kb) [file 12889_2017_4326_MOESM1_ESM.docx]

**Date Excel** ☐ **Email /post / both: PCF** ☐ **PIS** ☐

**Email / post / both: Interview conf.** ☐ **Etter** ☐

**Participant’s contact details**

Name

Address Postcode

Tel Mobile Email

**Recruitment strategy**

**Where did you hear about the study?**

[1] Facebook (status update)

[2] Twitter

[3] Facebook ad

[4] Flyer or word of mouth

[5] Media – talkback radio

[6] Media – print

[7] Other

**Personal information and demographics**

**Q1 Gender**

[1] Male

[2] Female

**Q2 What’s your date of birth?**

**Q3 What is the highest level of education you have attained?**

[1] No formal schooling

[2] Primary school

[3] Junior high school (Years 7-10)

[4] Senior high school (Years 11-12)

[5] TAFE/Technical college

[6] University OR

[7] Another tertiary institution

[8] Other (please specify)

**Q4 Which of the following best describes your employment status?**

[1] Working full time

[2] Working part-time or casual

[3] Retired

[4] Student

[5] Home duties

[6] Unemployed or looking for work

[7] Other (please specify)

**Q5 Roughly speaking, is your annual household income (before tax) more or less than $60,000?**

[1] Less than $60,000

[2] More than $60,000

**Q6 And into which of the following ranges would your annual household income fall?**

[1] Up to $15,000 ($290 per week)

[2] $15,001-$30,000 ($290-$580 per week)

[3] $30,001-$45,000 ($580-$860 per week)

[4] $45,001-$60,000 ($860-$1,150 per week)

[5] $60,001-$75,000 ($1,150-$$1,440 per week)

[6] $75,001-$90,000 ($1,440 -$1,730 per week)

[7] $90,001-$105,000 ($1,730-$2,020 per week)

[8] $105,001-$120,000 ($2,020-$2,300 per week)

[9] Over $120,000 ($2,300 per week)

**Screening questions – smoking status**

**Q1 Do you currently smoke cigarettes, cigars or pipes?**

⃝ No, not at all – go to Q2

[1] Yes – IF YES, how often?

[1] Daily – go to Q9

[1] At least weekly (if not daily) – go to Q9

[1] Less often than weekly – go to Q9

**Q2 Over your lifetime would you have smoked at least 100 cigarettes or a similar amount of tobacco?**

[2] No – EXCLUDE, THANK THEM FOR TAKING THE TIME TO ANSWER THE QUESTIONS

[3] Yes – go to Q3

**Q3 How frequently were you smoking?**

[1] Daily – go to Q4

[2] At least weekly (if not daily) – go to Q4

[3] Less often than weekly - go to Q4

**Q4 How many cigarettes per day / per week (or packs per day/ per week) were you smoking?**

[1] Fewer than 10 CPD (1/2 pack per day) - go to Q5

[2] More than 10 CPD (1/2 pack per day) – go to Q5

**Screening questions – time since quit and difficulty quitting**

**Q5a When did you give up smoking?**

Date Number of years/months ago

[1] Less than 6 months ago – EXCLUDE, THANK THEM FOR TAKING THE TIME TO ANSWER THE QUESTIONS

[2] More than 6 months ago but less than 2 years ago – go to Q6

[3] More than 2 years ago – EXCLUDE, THANK THEM FOR TAKING THE TIME TO ANSWER THE QUESTIONS

**Previous quit attempts**

**Q6 Was this the first time you tried to quit?**

[1] Yes – go to Q7

⃝ No – IF NO, how many times have you tried to quit before?

[2] Less than 3 – go to Q6b

[3] Between 3 and 10 – go to 6b

[4] More than 10 – go to Q6b

**Q6b On a scale of 1 to 10, if 1 is very easy and 10 is extremely difficult, how would you describe your last quit attempt?**

[1] [2] [3] [4] [5] [6] [7] [8] [9] [10] – go to Q6c

**Q6c Would you say that on your *last* quit attempt, quitting was:**

[1] Easier than you’d expected – go to Q7

[2] Harder than you’d expected – go to Q7

[3] Pretty much as you’d expected – go to Q7

**Screening questions – method of quitting**

**Q7 Thinking about [the LAST time you quit] OR [when you quit], would you say that you ‘gave up on your own’ or did anyone or anything help you to give up?**

[1] On my own – go to Q8

[2] With help ­­– go to Q8

**Q8 We’re interested in what people define as ‘giving up on their own’. I’d like to find out if you used any of the following strategies [the LAST time you quit] OR [when you quit].**

**8a Complementary or alternative therapies, such as hypnotherapy or acupuncture?**

[1] Yes – EXCLUDE, but go to Q8b

⃝ No – go to Q8b

**8b Incentive schemes that encourage people to give up smoking, such as a quit smoking competition or an incentive scheme at work?**

[1] Yes – EXCLUDE, but go to Q8c

⃝ No – go to Q8c

**8c Self-help materials such as brochures, books, CDs, DVDs, internet sites, apps for mobile devices, or the Quitline?**

⃝ No – go to Q8d

⃝ Yes – IF YES, did you use these self-help materials with the help or guidance of a health professional or counsellor? [DO NOT INCLUDE ‘BRIEF ADVICE’, i.e. VERBAL MESSAGE TO QUIT FROM DOCTOR OR NURSE]

[1] Aided by health professional or trained counsellor (includes using QuitCoach or follow-up support from Quitline) – EXCLUDE, but go to Q8d

⃝ Unaided by health professional or trained counsellor (includes calling Quitline or receiving Quitkit) – go to Q8d

**8d Counselling sessions, either one-on-one or in a group?**

[1] Yes – EXCLUDE, but go to Q8e

⃝ No – go to Q8e

**8e Nicotine replacement products that you can buy from chemists or supermarkets, such as gum, lozenges, tablets, inhalers or patches [Nicobate, Nicotinell, QuitX, Nicorette], or that you can buy online, such as e-cigarettes?**

[1] Yes – EXCLUDE, but go to Q8f

⃝ No – go to Q8f

**8f Prescription medications such as Champix [varenicline] or Zyban [bupropion], or nicotine replacement therapy patches [Nicobate, Nicotinell, QuitX, Nicorette]?**

[1] Yes – EXCLUDE, but go to Q8g

[2] No – INCLUDE, go to Q8g

**8g Are there any other forms of help that you used that I haven’t mentioned?**

FOR THOSE THAT MET THE SELECTION CRITERIA, ASK IF THEY’D BE INTERESTED IN FINDING OUT MORE ABOUT THE STUDY

**Additional questions to screen for ‘being serious’**

**Q9 What was it about the tweet or our website that made you decide to register your interest in the study?**

**Q10 Is there anything in particular about the study that interests you?**

**Q11 Do you believe ‘being serious’ is essential to quitting success?**

⃝ Yes

⃝ No
