# Supplementary material for: Measured, opportunistic, unexpected and naïve quitting: a qualitative grounded theory study of the process of quitting from the ex-smokers’ perspective
Source: BMC Public Health. 2017 May 11;17:430. doi: 10.1186/s12889-017-4326-4 (PMC5426051; doi:10.1186/s12889-017-4326-4)
Supplement: Supplementary file 2 — Interview questions. The interview schedule listing the type of questions asked in the semi-structured interviews. (DOC 40 kb) [file 12889_2017_4326_MOESM2_ESM.doc]

**Natural history of unassisted smok**ing cessation – Interview questions

| **Questions** | **Prompts** |
| --- | --- |
| **1. Smoking history**  Tell me about when you first started smoking.  *[Interviewee will be asked to draw on a piece of paper a timeline beginning with when they started smoking]* | - Age when first took up smoking - Reasons for starting to smoke - Amount smoked - Situations in which they smoked |
| **2. Quitting history**  Have you ever tried to quit before?  Show me on this timeline when you had a go at giving up smoking, no matter how short.  *[Indicate year/age when interviewee stopped and for how long; ask participant to include significant milestones on timeline, such as leaving home, getting married, having children as an aid to prompt recall of quit attempts].*  Tell me a bit about each of the times you quit.  *[For each quit attempt use prompts in next column as appropriate.]* | For each quit attempt:  **Deciding to quit**   - Describe the events that led up to you quitting - Tell me about anything that might have had an influence on your decision to quit - Had you been planning on quitting?   **Deciding *how* to quit**   - Tell me about how you quit – did you quit on your own, did someone help you or did you use anything to help you quit? What help did you use? - Tell me about why you decided to quit on your own/use [insert type of assistance] - Tell me about the attitudes or reactions of others to your decision to quit on your own/using [insert type of assistance]. Did anything anyone say or do affect your decision to quit or your quit attempt?   **The quitting experience**   - Tell me about your experience of quitting on your own/using [insert type of help]. Was it harder or easier than you had expected? - How did this quit attempt compare with your experience of previous quit attempts? - Tell me about anything that helped your quit attempt or hindered your quit attempt - How did your attempts to quit differ when you used [insert type of help] and when you tried to quit on your own? - Tell me about when you started smoking again. Why do you think you started smoking again?   **For assisted quit attempts**   - How did you think the [insert type of assistance] would help? How did this compare with your experience with it? |
| **3. Unassisted quitter’s toolbox**  Imagine I’m about to stop smoking today. What advice would you give me about how I should quit?  If I decided to quit on my own, what advice would you give me? | **Strategies/techniques used to help quit unassisted**   - Tell me about any situations or experiences that made quitting difficult? Or any situations that helped you to quit? - Who was most helpful to you during this time? How was he/she helpful?   **Barriers to quitting**   - social (e.g. friends who smoke) - psychological (e.g. stress relief)   **Facilitators of quitting**   - environmental (e.g. workplace smoking bans) - structural (e.g. tax increases) - personal (e.g. health, pressure from family, image)   **Strategies/techniques to prevent relapse**   - Tell me about any situations or experiences that make staying quit difficult? Or any situations that have helped you to stay quit? - Do you have any strategies/tricks to help out when you feel tempted to smoke? - Did your previous quitting experience affect how you handled your final successful quit attempt? - Do you think you will ever start smoking again? Tell me about that. |
